# Long-Term Outcomes in Real Life of Lumacaftor–Ivacaftor Treatment in Adolescents With Cystic Fibrosis

**DOI:** 10.3389/fped.2021.744705

**Published:** 2021-11-15

**Authors:** Stéphanie Bui, Alexandra Masson, Raphaël Enaud, Léa Roditis, Gaël Dournes, François Galode, Cyrielle Collet, Emmanuel Mas, Jeanne Languepin, Michael Fayon, Fabien Beaufils, Marie Mittaine

**Affiliations:** ^1^Bordeaux University Hospital, Hôpital Pellegrin-Enfants, Paediatric Cystic Fibrosis Reference Center (CRCM), Centre d'Investigation Clinique (CIC 1401), Bordeaux, France; ^2^Limoges University Hospital, Paediatric Cystic Fibrosis Reference Center (CRCM), Limoges, France; ^3^Bordeaux University, Centre de Recherche Cardio-Thoracique de Bordeaux, U1045, Radiology, Bordeaux, France; ^4^Toulouse University Hospital, Paediatric Cystic Fibrosis Reference Center (CRCM), Department of Pediatric-pulmonology, Toulouse, France

**Keywords:** cystic fibrosis, child, CFTR potentiator, CFTR corrector, lung function testing, nutritional status, sweat chloride

## Abstract

**Background:** The combination of the CFTR corrector lumacaftor (LUM) and potentiator ivacaftor (IVA) has been labeled in France since 2015 for F508del homozygote cystic fibrosis (CF) patients over 12 years. In this real-life study, we aimed (i) to compare the changes in lung function, clinical (e.g., body mass index and pulmonary exacerbations) and radiological parameters, and in sweat chloride concentration before and after initiation of LUM/IVA treatment; (ii) to identify factors associated with response to treatment; and (iii) to assess the tolerance to treatment.

**Materials and Methods:** In this tri-center, non-interventional, and observational cohort study, children (12–18 years old) were assessed prospectively during the 2 years of therapy, and retrospectively during the 2 years preceding treatment. Data collected and analyzed for the study were exclusively extracted from the medical electronic system records of the patients.

**Results:** Forty adolescents aged 12.0–17.4 years at LUM/IVA initiation were included. The lung function decreased significantly during and prior to treatment and increased after LUM/IVA initiation, becoming significant after 2 years of treatment. LUM/IVA significantly improved the BMI *Z*-score and sweat chloride concentration. By contrast, there was no significant change in exacerbation rates, antibiotic use, or CT scan scores. Age at LUM/IVA initiation was lower in good responders and associated with greater ppFEV1 change during the 2 years of treatment. LUM/IVA was well-tolerated.

**Conclusion:** In F508del homozygote adolescents, real-life long-term LUM/IVA improved the ppFEV1 trajectory, particularly in the youngest patients, nutritional status, and sweat chloride concentration but not exacerbation rates or radiological scores. LUM/IVA was generally well-tolerated and safe.

## Introduction

Cystic fibrosis (CF) is an inherited genetic disease leading to *cystic fibrosis transmembrane conductance regulator* (CFTR) dysfunction. CFTR is involved in active excretion of chloride at the apical membrane of the epithelial cells (1–4). Among more than 2,000 mutations of the CFTR gene, the homozygous F508del represents the most frequent in France with a prevalence of up to 41% of patients (5). This mutation leads to a processing and trafficking defect of the CFTR protein resulting in an early degradation or a dysfunctional protein expressed into the apical membrane of the cells (1, 4, 6). As a consequence, epithelial mucus secretion is dehydrated, promoting pulmonary infection (7) and organ injury (e.g., pancreatic insufficiency), leading to impaired lung function, growth, and nutritional outcomes (8–10). Therapeutic approaches over the last 20 years based on symptomatic treatments (physiotherapy, fluidifiers, and pancreatic enzymes, etc.) (11, 12) slowed down the progression of CF and enhanced life expectancy (4, 10). Nevertheless, CF remains a severe disease, in which pulmonary exacerbations and poor nutritional status contribute to progressive lung function decline and death in young adults (4, 8, 10). The highest decline in lung function occurs during adolescence which is thus a pivotal period in the management of the disease (13).

In this context, therapeutic approaches have been developed to improved cell chloride secretion by targeting the defect in F508del-CFTR folding using in association: the CFTR corrector lumacaftor (LUM) (enhances trafficking and processing), and the CFTR potentiator ivacaftor (IVA) (increases the channel opening probability) (1, 4). Phase III studies have demonstrated good tolerance of the lumacaftor/ivacaftor association (LUM/IVA) and significant potential benefits in young children aged 2–5 years old (14) and 6–11 years (15, 16) [e.g., improved body mass index (BMI)], and in adolescents over 12 years of age and adults [i.e., improvement in BMI and lung function, assessed by the percentage of predicted forced expiratory volume in 1 s (ppFEV1), and decrease in the number of exacerbations usually defined by clinical deterioration treated with IV antibiotics] (17). The positive effect of LUM/IVA on lung function decline and BMI has been confirmed in patients over 12 years by the 96 weeks follow-up PROGRESS study (8) and more recently, in France, in the real-life study performed by Burgel et al. (18). However, the PROGRESS study mostly included CF adults (8), and the study performed by Burgel et al. followed CF adolescents only for a year after LUM/IVA initiation.

Thus, real-life studies are needed to confirm the ability of LUM/IVA to change the course of the disease and to evaluate its long-term effects, particularly in adolescents, who are at a critical stage of their development.

Thus, we conducted a pediatric prospective real-life cohort study including F508del homozygote adolescents followed-up within the French South-Western network (MUCOSUD) to assess the evolution of the ppFEV1 before and after the LUM/IVA initiation. The secondary objectives were to assess (i) the evolution of clinical [i.e., anthropometric parameters (weight, height, and BMI expressed as *Z*-scores) and number of pulmonary exacerbation, antibiotic use], biological (i.e., sweat chloride concentration), and radiological (i.e., the Bhalla CT-scan score with the percentage high attenuation volume (%HAV) mucus secretions sub-score) parameters; (ii) to identify parameters associated with response to treatment; and (iii) to assess the tolerance to treatment.

## Methods

The study was conducted prospectively between February 2016 and December 2018 within the French South-West network of pediatric CF centers (tertiary-care university hospitals in Bordeaux, Toulouse, and Limoges) and the included CF adolescents (12–18 years old) eligible for LUM/IVA treatment (F508del homozygotes aged 12 years and older) not included in other CFTR modulator protocols study. Patients having stopped LUM/IVA within 3 months after initiation of treatment were excluded from the analyses. It was a non-interventional study. Indeed, LUM/IVA has been approved by the Food and Drug Administration and the European Medicines Agency, and labeled in France since 2015 for CF patients with F508del homozygous CFTR mutation over 12 years of age. Thus, at the time of the patient follow-up period, LUM/IVA was prescribed as part of the routine care of a patient. In addition, the use of the data collected and analyzed for the study were exclusively extracted from the medical records of the patients (MUCODOMEOS, https://www.vaincrelamuco.org/2019/05/09/mucodomeos-un-logiciel-adapte-aux-besoins-des-crcm-2684) after having obtained their informed consent. In this context and according to the French law in force, the approval of an ethics committee was not required.

### Data Collected

**Figure 1:**
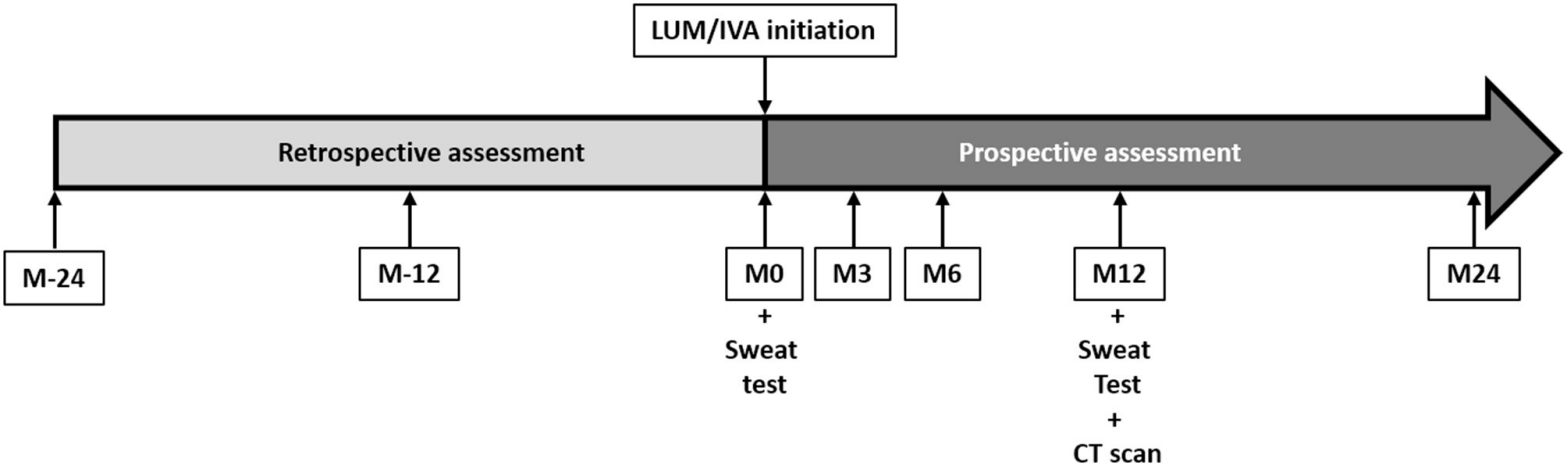
Study design and chronology. Patients were included at the initiation of lumacaftor/ivacaftor (LUM/IVA) (M0). At 3 (M3), 6 (M6), 12 (M12), and 24 (M24) months of treatment, height, weight, body mass index, and lung function testing results were assessed prospectively. These parameters were also assessed retrospectively at 24 (M-24) and 12 (M-12) months before the LUM/IVA initiation. A sweat test was performed at M0 and M12. Computed tomography scans were performed at M12. The side effects of LUM/IVA treatment were assessed at each visit after initiation.

All children were followed prospectively at least every 3 months before and after the LUM/IVA initiation as part of their disease follow-up (Figure 1). Briefly, weight, height, BMI, and ppFEV1 were assessed the day of the LUM/IVA initiation (M0) and at 3, 6, 12, and 24 months (M3, M6, M12, and M24) after the treatment initiation during routine clinical visits of the patients. To assess the effect of the LUM/IVA treatment on the disease course, theses parameters were retrospectively collected 12 and 24 months (M-12 and M-24) prior to the initiation of LUM/IVA. Anthropometric data (weight, height, and BMI) were automatically transformed as *Z*-scores by the MUCODOMEOS software based on the World Health Organization standardized values (19). The ppFEV1 was determined using the Global Lungs Initiative 2012 reference values. Before treatment, we chose retrospectively the best ppFEV1 value for each study date (±1 month). At initiation and during the 2 years of follow-up, the FEV1 was collected prospectively at each study visit. Good responders were defined as a ppFEV1 gain >5% at M24 compared to M0. The 5% threshold was chosen in accordance with previous studies (17, 20, 21). Adolescence is the period with the highest decline in lung function (14), and improvement in ppFEV1 is an “optimal” outcome. However, having a stable lung function may be considered a success. We therefore defined among the patients who were not good responders to 2 other groups: the no-decline patients characterized by a ppFEV1 gain between 0 and 5%, and the low/non-responders characterized by a decrease ppFEV1 at M24 compared to M0. To evaluate the evolution of the response to treatment of the patients, the presence of a good response, no decline response, or low/no response was evaluated by the ppFEV1 change at M3, M6, and M12 (vs. M0) using the same thresholds. The number of oral and/or intravenous antibiotics courses and the number of exacerbations [i.e., acute pulmonary exacerbation requiring oral or intravenous antibiotic therapy as defined previously for clinical research (22)] that occurred in the 2 years following treatment initiation and in the 2 years prior to treatment were collected. A sweat test was performed at M0 and M12 according to European recommendations (23).

CT scans performed in the radiology department of the inclusion centers before and after 1 year of treatment were centralized for the study evaluations. Two evaluations were performed: a visual CT Bhalla score (24) and an automated measurement of percentage high attenuation volume (%HAV) (25). Visual scorings were performed independently by two radiologists from Bordeaux University Hospital, with previous publication experience in CT scoring in CF (26–28). The means of these evaluations were kept for further analyses. The %HAV was automatically measured by using the Pulmo3D Syngo software (Siemens, Erlangen, Germany).

To assess treatment tolerance, plasma liver enzymes were monitored prior to the LUM/IVA treatment initiation and at each visit during the first year and annually thereafter. Eye examinations were performed prior to the start of treatment and monitored at 1 year. At each visit, we also collected clinical data regarding side effects and recorded adherence to treatment in order to avoid a lack of response due to poor adherence treatment.

### Statistics

Analyses were performed only on available data using R software® (version 4.0). Figures were done using Prism software® (version 5.1). Data from the entire population were analyzed and comparison between subgroups according to the presence or absence of impaired lung function (ppFEV1 < 80%) or according to the *Z*-scores BMI (*Z*-scores < 0 vs. *Z*-scores ≥ 0) at the LUM/IVA initiation) were performed. Normality was assessed using Q-Q plots. Parametric variables were compared using Mann–Whitney test or ANOVA, and results were expressed as mean ± standard deviation (SD). Non-parametric variables were compared with Mann–Whitney test for unpaired values or Wilcoxon test for paired values and with *t*-test for parametric variables. Results were expressed as the mean ± standard deviation or as the median and interquartile range [median (IQR_25_; IQR_75_)]. Categorical variables were expressed as absolute values and as percentages. Categorial variables were performed using the Fisher's exact test or Chi square test. Correlations were performed using the Spearman test. A *p*-value < 0.05 was considered significant.

## Results

### Population Characteristics

**Figure 2:**
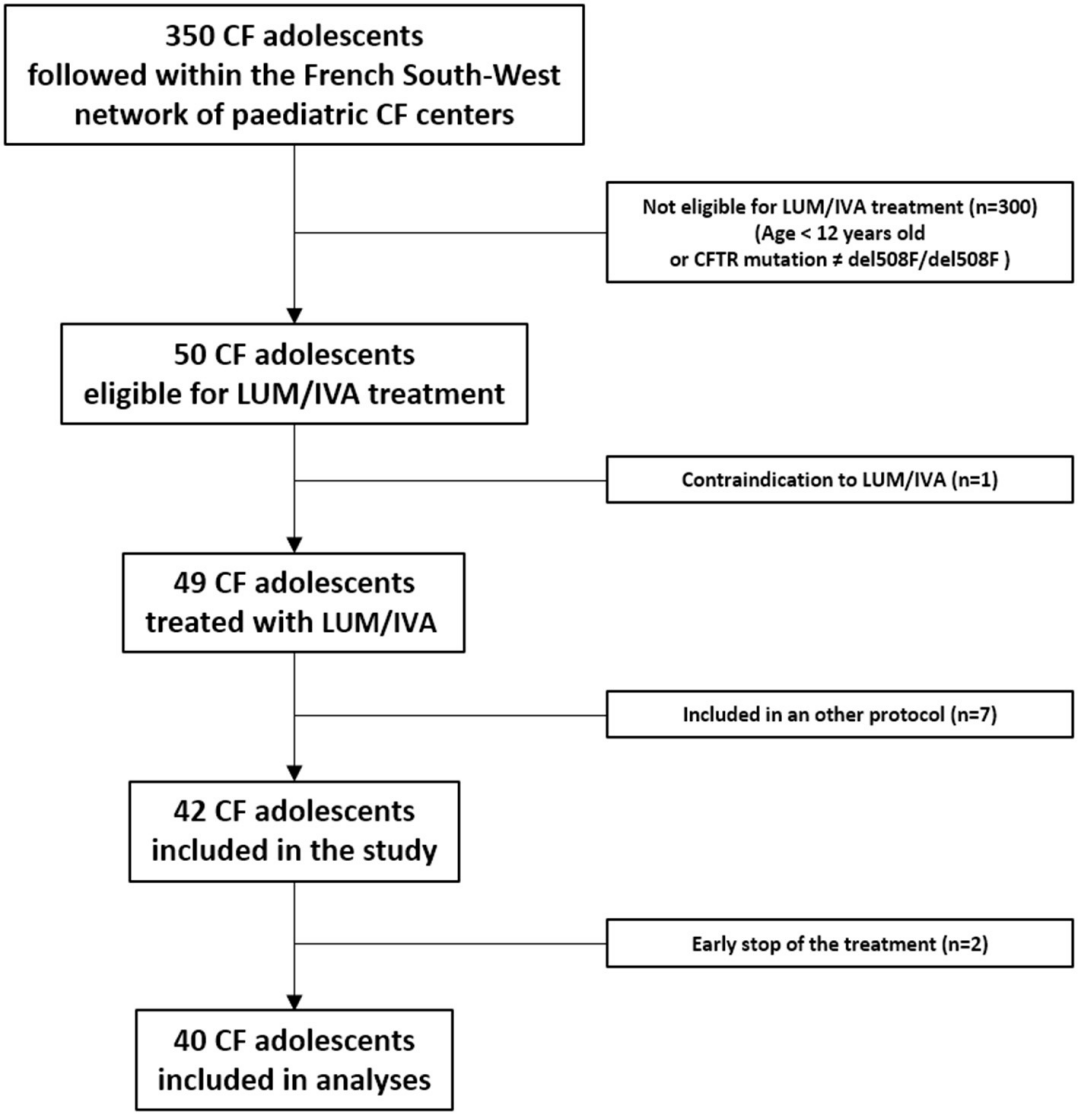
Flow chart. CF, cystic fibrosis; LUM/IVA, lumacaftor/ivacaftor association; del508F/del508F, homozygous deletion of the codon for phenylalanine at position 508 in the cystic fibrosis transmembrane regulator (CFTR) gene.

Among the 350 adolescents with CF followed within the French South-West network of pediatric CF centers (MUCOSUD), 50 were eligible for the LUM/IVA treatment of whom one had a contraindication for LUM/IVA (severe cirrhosis) and seven were included in new therapies clinical trials (Figure 2). LUM/IVA was initiated in the remaining 42 included patients but two discontinued the LUM/IVA treatment early (<3 months) and were excluded from the analyses (Figure 2).

The main characteristics of the 40 adolescents at initiation of LUM/IVA are summarized in Table 1. At M0, there was a negative correlation between sweat chloride and both ppFEV1 (*r* = −0.46; *p* = 0.009) and BMI Z-scores (*r* = −0.42; *p* = 0.022), and a positive correlation between ppFEV1 and BMI Z-score (*r* = 0.46; *p* = 0.004). Except for lower ppFEV1 and BMI *Z*-scores in patients with impaired lung function (ppFEV1 < 80%) at M0, there was no significant difference between patients according to the presence or the absence of impaired lung function at M0 (Table 1). Except for anthropometric parameters (i.e., weight, height, and BMI *Z*-scores) and sweat chloride, there was no difference between patients with or without BMI *Z*-scores < 0 at M0 (Table 1).

**Table 1:** Characteristics of patients at lumacaftor/ivacaftor initiation.

	**Total**	**ppFEV1 <80% at M0**	**ppFEV1 ≥80% at M0**	***p*1**	**BMI *Z*-score <0 at M0**	**BMI *Z*-score ≥0 at M0**	***p*2**
*N*	40	18	22		24	16	
Age (years)	13.9 ± 1.7	13.5 ± 1.8	14.2 ± 1.5	0.111	13.8 ± 1.7	14.0 ± 1.8	0.814
Male	22/40 (55.0)	8/18 (44.4)	14/22 (63.6)	0.339	14/24 (58.3)	8/16 (50.0)	0.748
Weight *Z*-score	−0.40 ± 1.01	−0.71 ± 1.02	−0.15 ± 0.94	0.134	−1.00 ± 0.70	0.51 ± 0.63	**<0.001**
Height *Z*-score	−0.37 ± 1.06	−0.41 ± 1.17	−0.34 ± 0.99	0.860	−0.66 ± 1.07	0.06 ± 0.91	**0.030**
BMI *Z*-score	−0.28 ± 1.00	−0.68 ± 0.97	0.04 ± 0.94	**0.036**	−0.92 ± 0.61	0.75 ± 0.52	**<0.001**
Past history							
Meconial ileus	2/40 (5.0)	1/18 (5.6)	1/22 (4.5)	1.000	1/24 (4.2)	1/16 (6.3)	1.000
DIOS	1/40 (2.5)	0/18 (0.0)	1/22 (4.5)	1.000	0/24 (0.0)	1/16 (6.3)	0.400
CFRD	2/40 (5.0)	0/18 (0.0)	2/22 (9.1)	0.492	1/24 (4.2)	1/16 (6.3)	1.000
CFLD	9/40 (22.5)	6/18 (33.3)	3/22 (13.6)	0.253	7/24 (29.2)	2/16 (12.5)	0.272
*P. Aeruginosa* colonization							
None	18/40 (45.0)	7/18 (38.9)	11/22 (50.0)		10/24 (41.7)	8/16 (50.0)	
Intermittent	11/40 (27.5)	5/18 (27.8)	6/22 (27.3)	0.713	8/24 (33.3)	3/16 (18.8)	0.598
Chronic	11/40 (27.5)	6/18 (33.3)	5/22 (22.7)		6/24 (25.0)	5/16 (31.3)	
*S.Aureus* colonization							
None	8/40 (20.0)	4/18 (22.2)	4/22 (18.2)		5/24 (20.8)	3/16 (18.8)	
Intermittent	3/40 (7.5)	0/18 (0.0)	3/22 (13.6)	0.264	1/24 (4.2)	2/16 (12.5)	0.618
Chronic	29/40 (72.5)	14/18 (77.8)	15/22 (68.2)		18/24 (75.0)	11/16 (68.8)	
ppFEV1	83.3 ± 18.3	68.2 ± 12.7	95.6 ± 11.7	**<0.001**	80.1 ± 18.2	88.1 ± 18.0	0.109
Sweat chloride (mmol/L)	104.7 ± 16.6	99.2 ± 17.8	110.3 ± 14.8	0.189	110.1 ± 14.2	97.3 ± 17.4	**0.039**
Treatment							
PERT U/Kg/j	7302 ± 1727	7554 ± 1887	7107 ± 1609	0.506	7741 ± 1738	6600 ± 1507	0.116
Proton pump inhibitor	21/40 (52.5)	9/18 (50.0)	12/22 (54.5)	1.000	13/24 (54.2)	8/16 (50.0)	1.000
Ursodeoxycolic acid	16/40 (40.0)	7/18 (38.9)	9/22 (40.9)	1.000	10/24 (41.7)	6/16 (37.5)	1.000
Laxative treatment	5/40 (12.5)	0/18 (0.0)	5/22 (22.7)	0.053	1/24 (4.2)	4/16 (25.0)	0.138
Inhaled antibiotic	17/40 (42.5)	10/18 (55.6)	7/22 (31.8)	0.200	10/24 (41.7)	7/16 (43.8)	1.000

*M0, day of lumacaftor/ivacaftor initiation; ppFEV1, percent of predicted forced expiratory volume in 1 s; BMI, body mass index; DIOS, distal intestinal obstruction syndrome; CFRD, cystic fibrosis related diabetes; CFLD, cystic fibrosis liver disease; P. Aeruginosa, Pseudomonas Aeruginosa; S. Aureus, Staphylococcus Aureus; PERT, pancreatic enzymes replacement therapy*.

*Results are expressed as n/N (%) for categorical variables or as mean ± standard deviation for quantitative variables. Comparisons between patients with ppFEV1 < 80% compared with those with ppFEV1 ≥ 80% at M0 (p1) or between (patients with BMI Z-score < 0 compared with those with BMI Z-score ≥ 0 at lumacaftor/ivacaftor initiation (p2) were performed using the Fisher's exact test for categorical variables or the Mann–Whitney test for quantitative variables. A p-value ≤ 0.05 was considered significant*.

### Lumacaftor/Ivacaftor Changes the Trajectory of Both ppFEV1 and BMI *Z*-Score and Improves Sweat Chloride in Adolescents With Cystic Fibrosis

**Figure 3:**
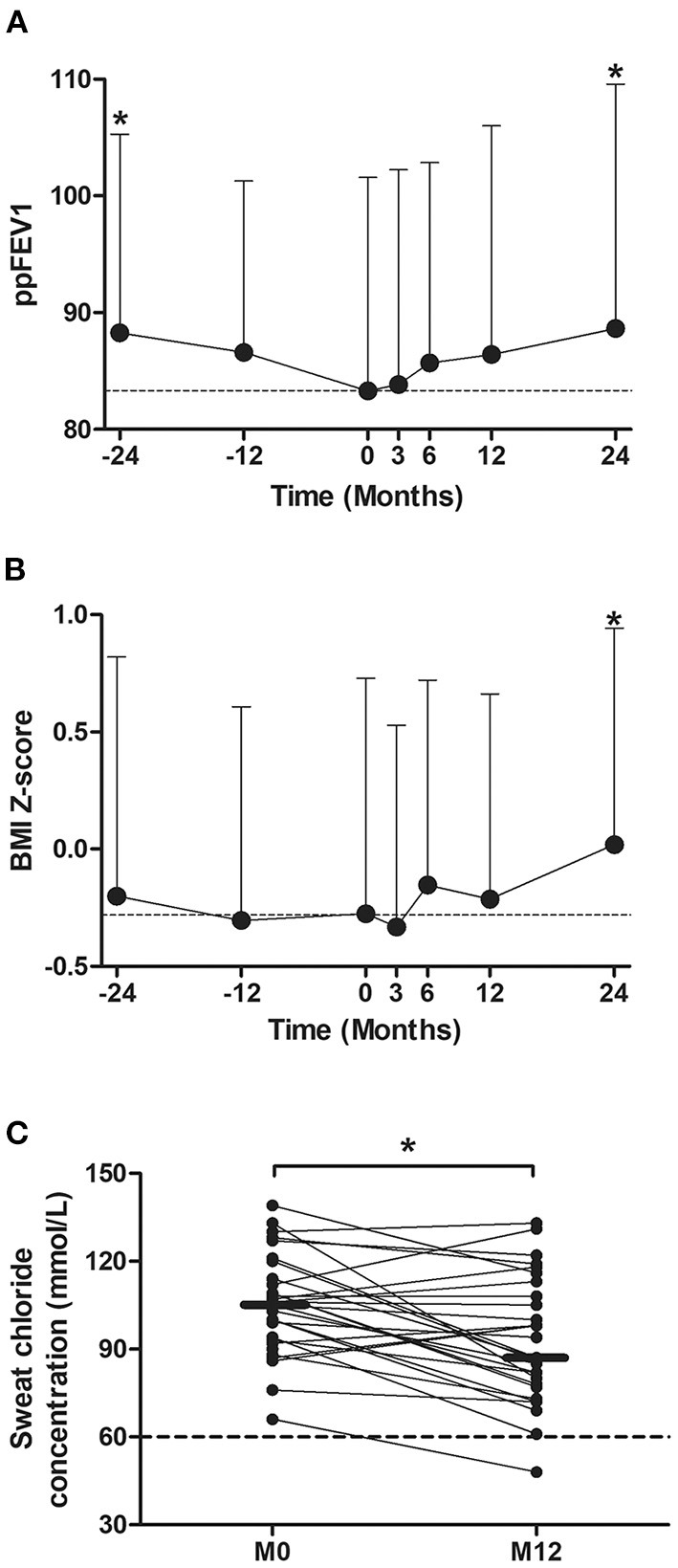
Evolution of the percent predicted forced expiratory volume in 1 s (ppFEV1), body mass index (BMI) *Z*-score, and sweat chloride before and after the initiation of lumacaftor/ivacaftor (LUM/IVA). Evolution of ppFEV1 **(A)** and BMI *Z*-score **(B)** from 2 years before to 2 years after lumacaftor/ivacaftor (LUM/IVA) initiation (M0). Evolution of sweat chloride from M0 to 12 months after lumacaftor/ivacaftor (LUM/IVA) initiation **(C)**. Data are plotted at each timepoint using all available and were represented at the mean ± standard deviation **(A,B)**. Comparisons with data obtained at M0 were performed using the Wilcoxon paired test (*p < 0.05).

From 2 years before (M-24) to LUM/IVA initiation (M0), the ppFEV1 decreased significantly from 88.3% ± 17.0 to 83.4% ± 16.5 (*p* = 0.008) (Figure 3A). By contrast, 2 years after LUM/IVA initiation (M24), the ppFEV1 had increased significantly to 89.2% ± 20.9 corresponding to an absolute change of +5.8% ± 12.1 compared with M0 (*p* = 0.027) (Figure 3A). To note, the ppFEV1 measured at M3, M6, and M12, as well as at M-12, were not significantly different compared with that measured at M0 (Figure 3A). In addition, the BMI *Z*-score was stable from M-24 to M0 (*p* = 0.28), increased after the LUM/IVA initiation with a significant improvement to 0.02 ± 0.92 at M24 corresponding to an increase of 0.31 ± 0.66 compared with M0 (*p* = 0.006) (Figure 3B). Interestingly, the weight *Z*-score (Supplementary Figure 1A) but not the height *Z*-score (Supplementary Figure 1B) increased significantly between M0 and M24. Moreover, sweat chloride improved significantly from the LUM/IVA initiation to M12 (*p* = 0.002) but remained higher than the upper limit of the normal (Figure 3C). Surprisingly, we did not find any significant difference in either the number of exacerbations, or oral or intravenous antibiotic use in the 2 years before and after the LUM/IVA initiation (Supplementary Figures 2A–C). We also did not find difference in the Bhalla or HAV scores in the 24 patients who underwent a CT scan before the start and after 1 year of the LUM/IVA initiation (Supplementary Figures 3A,B).

### Subgroup Analyses of the Effect of Lumacaftor/Ivacaftor on Changes in ppFEV1, BMI *Z*-Score, and Sweat Chloride

**Figure 4:**
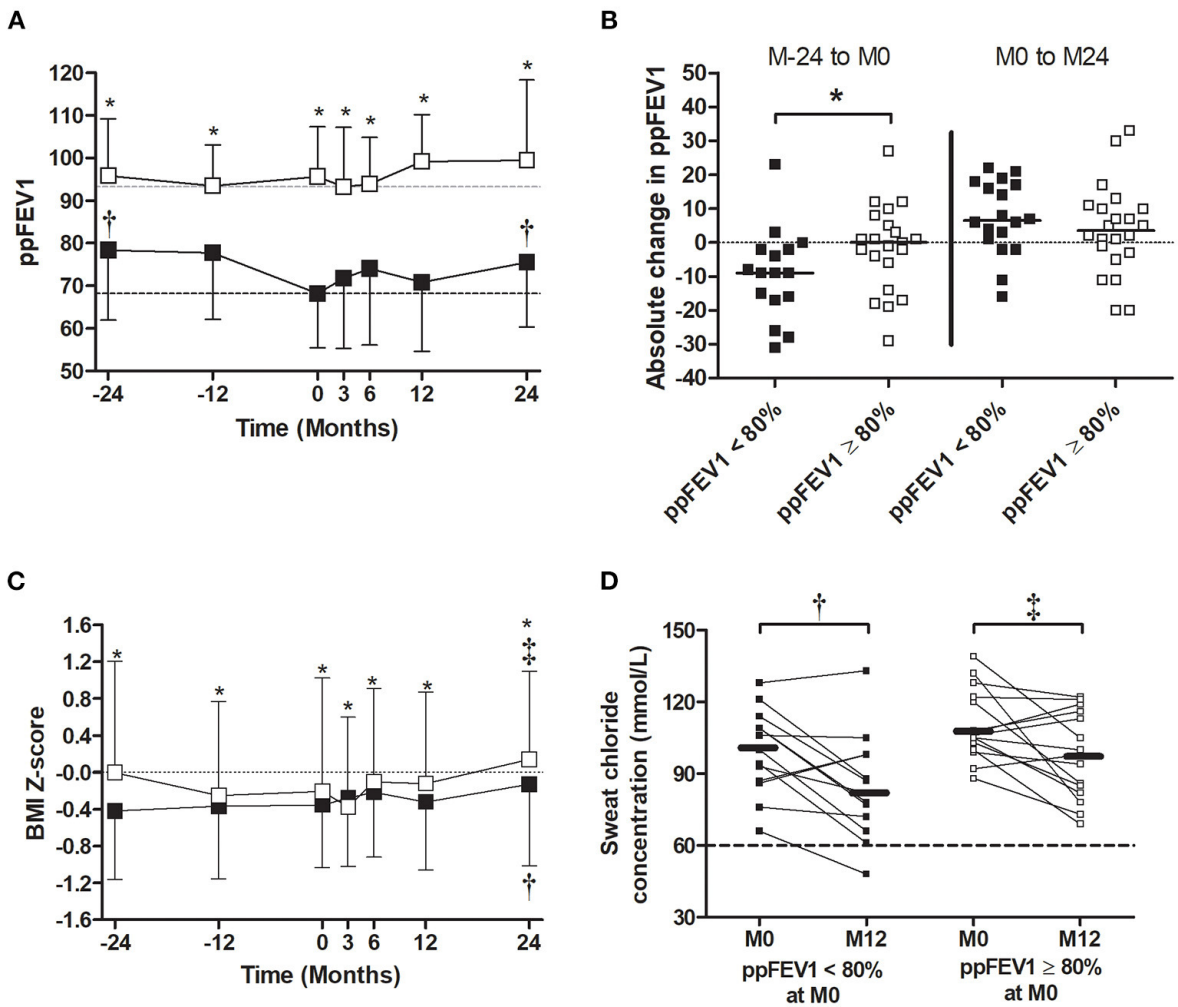
Evolution of the percent predicted forced expiratory volume in 1 s (ppFEV1), body mass index (BMI) *Z*-score, and sweat chloride before and after the initiation of lumacaftor/ivacaftor (LUM/IVA) in patient with or without ppFEV1 <80%. Evolution of ppFEV1 **(A)** and BMI *Z*-score **(C)** 2 years prior (M-24) to 2 years after (M24) LUM/IVA initiation (M0) in patient with (black square) or without (empty square) ppFEV1 < 80%. Absolute changes of ppFEV1 between M-24 to M0 [**(B)** left] and M0 to M24 [**(B)** right]. Evolution of sweat chloride from M0 to M12 in patients with or without lung function **(D)**. Comparisons with data obtained at M0 were performed using the Wilcoxon paired test **(A,B,D)** and between the two groups using the Mann–Whitney test. *, ^†^, and ^‡^Indicate significant difference (*p* < 0.05) compared with M0 in patients with ppFEV1 < 80% (^†^) and in patients with ppFEV1 ≥ 80% (^‡^) or between groups (*).

In patients with impaired lung function (ppFEV1 < 80%) at the LUM/IVA initiation, the ppFEV1 increased significantly from M0 to M24 (ppFEV1 absolute change of +7.3 ± 10.8%) after a significant decrease prior to the LUM/IVA initiation (ppFEV1 absolute change of −9.4 ± 13.3%) (Figure 4A). The same trend was observed in patients without impaired lung function at M0, but the difference was not significant (Figure 4A). Of note, the ppFEV1 was already lower at M-24 prior to the start of LUM/IVA and remained lower during the study period in patients with ppFEV1 < 80% at M0 compared with those with ppFEV1 ≥ 80% (Figure 4A). In addition, the decrease in ppFEV1 in the 2 years prior to the LUM/IVA initiation was greater in patients with impaired lung function than those without at M0 (Figure 4B); however, the ppFEV1 change from M0 to M24 was not different between the two groups (Figure 4B) as well as between M-24 and M24 (ppFEV1 change: −2.8 ± 17.9 vs. 3.4 ± 15.8, *p* = 0.086). Prior to treatment, the BMI *Z*-score remained stable in the two groups (Figure 4C). After the LUM/IVA initiation, in both groups of patients (with or without impaired lung function), the BMI *Z*-score increased, with a significant improvement at M24, but remained significantly lower in patients with BMI *Z*-score < 0 at M0 vs. the others, from M-24 to M24 (Figure 4C). In the two groups, the sweat chloride decreased significantly after a year of treatment (Figure 4D).

**Figure 5:**
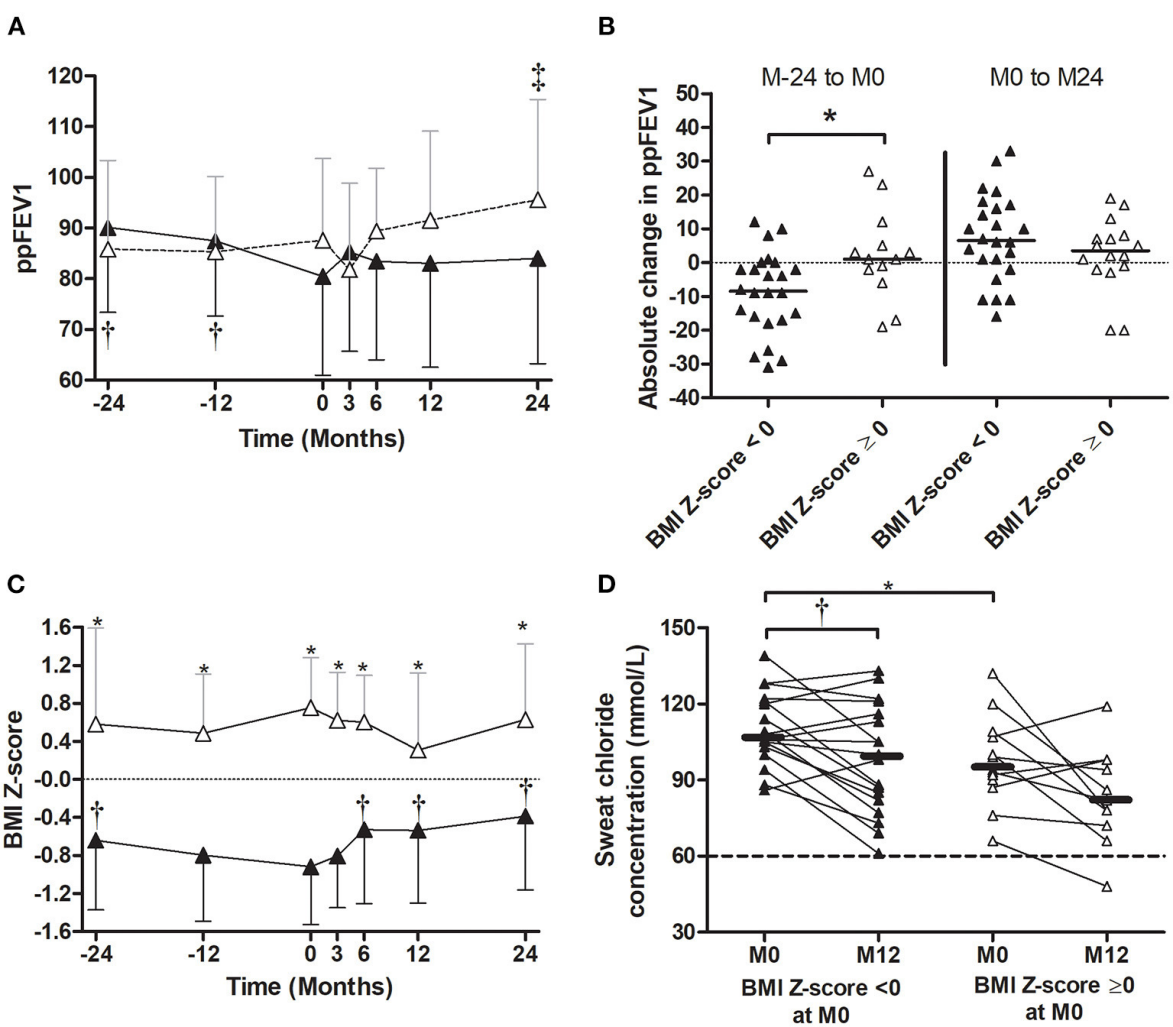
Evolution of the percent predicted forced expiratory volume in 1 s (ppFEV1), body mass index (BMI) *Z*-score, and sweat chloride before and after the initiation of lumacaftor/ivacaftor (LUM/IVA) in patient with or without BMI *Z*-score < 0. Evolution of ppFEV1 **(A)** and BMI *Z*-score **(C)** 2 years prior (M-24) to 2 years after (M24) LUM/IVA initiation (M0) in patient with (black triangle) or without (empty triangle) BMI *Z*-score < 0. Absolute changes of ppFEV1 between M-24 to M0 [**(B)** left] and M0 to M24 [**(B)** right]. Evolution of sweat chloride from M0 to M12 in patients with or without BMI *Z*-score < 0 (D). Comparisons with data obtained at M0 were performed using the Wilcoxon paired test **(A,B,D)** and between the two groups using the Mann–Whitney test. *, ^†^, and ^‡^ Indicate significant difference (*p* < 0.05) compared with M0 in patients with ppFEV1 < 80% (^†^) and in patients with ppFEV1 ≥ 80% (^‡^) or between groups (*).

In patients with a BMI *Z*-score < 0 at the LUM/IVA initiation, the ppFEV1 decreased significantly in the 2 years prior to the LUM/IVA initiation but remained stable in the following 2 years (Figures 3C,D), whereas in patients with BMI *Z*-score > 0 at M0, the ppFEV1 did not change before the LUM/IVA initiation and increased in the following 2 years with a significant improvement at M24 (Figure 5A). Indeed, the decrease in ppFEV1 in the 2 years prior to the LUM/IVA initiation was greater in patients with BMI *Z*-score < 0 than at M0 for those without (Figure 5B), whereas the increase in ppFEV1 from M0 to M24 was not different between the two groups (Figure 5B). The BMI *Z*-score decreased significantly between M-24 and M0 in patients with a BMI *Z*-score < 0 at the LUM/IVA initiation and increased significantly after as early as 6 months of treatment (Figure 5C). In contrast, in patients with a BMI *Z*-score ≥ 0 at the LUM/IVA initiation, the BMI *Z*-score did not change significantly from M-24 to M24 (Figure 5C). A decrease in sweat chloride was observed in both patients with and those without a BMI *Z*-score < 0, but the difference was only significant for the first group (Figure 5D).

### The Number of Good Responders Increased in Time and Was Associated With Age at the Lumacaftor/Ivacaftor Initiation

**Figure 6:**
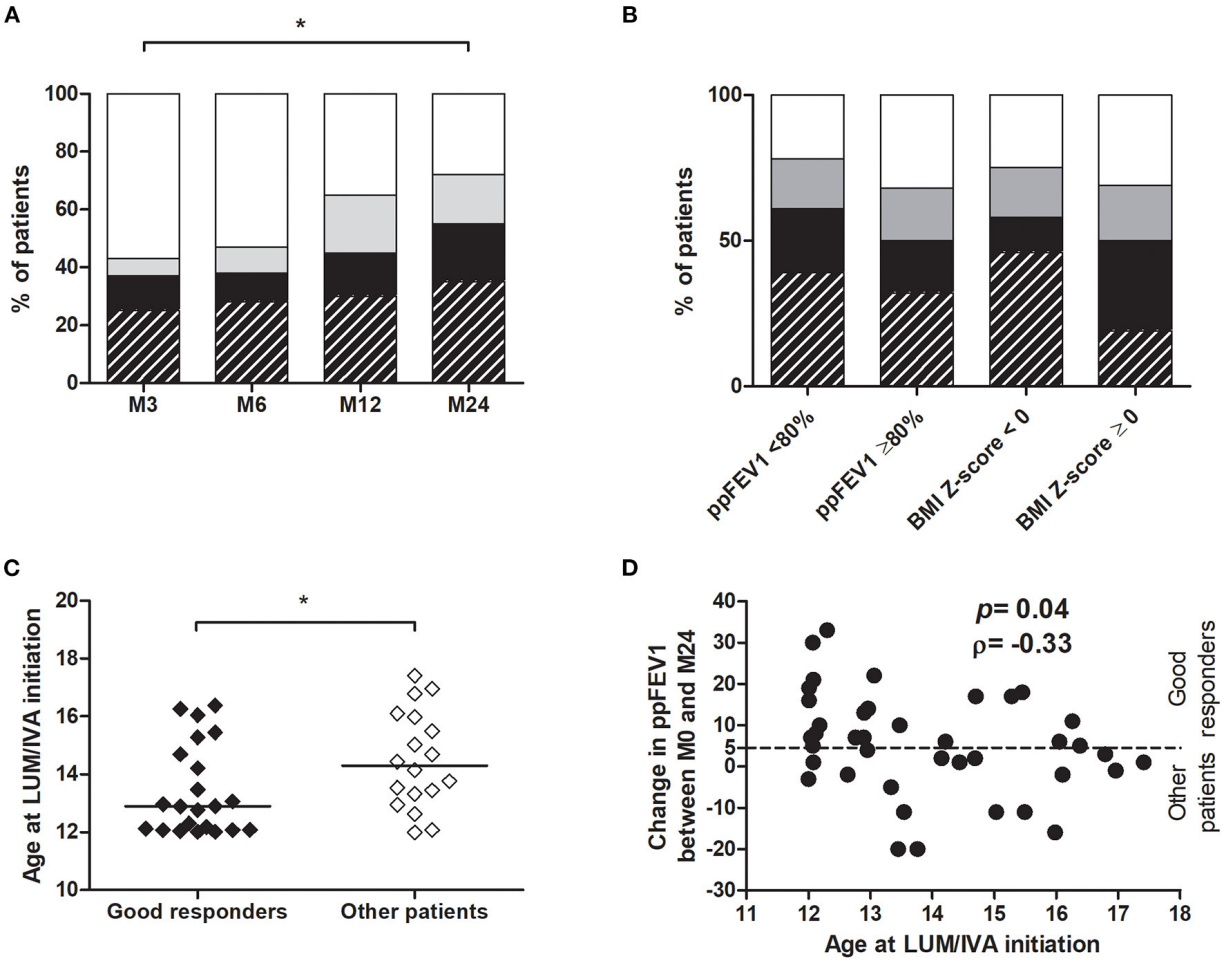
Evaluation of LUM/IVA treatment response over time and factors associated with a good response. For each time point, responses of patients were evaluated by the ppFEV1 change at the time point compared with M0 **(A,B)**. Patients with a good response to treatment (ppFEV1 change ≥5%) are represented in hatched black (ppFEV1 change >10%) or black (ppFEV1 change >5%), those in the no decline group (ppFEV1 change between 0 and 5%) in gray and the low/non responders (ppFEV1 decline) in white **(A,B)**. Comparisons between good responders (hatched black and black) and other patients at 3 (M3), 6 (M6), 12 (M12), and 24 months (M24) after LUM/IVA initiation was performed using the Chi-square test **(A,B)**. Age at initiation of LUM/IVA was compared between good responders, defined by their ppFEV1 change >5% at M24, and other patients (no decline group + low/non responders) **(C)**. Comparisons between groups were performed using the Mann–Whitney test. *Indicates *p* < 0.05. Correlation between age at LUM/IVA initiation and ppFEV1 change between LUM/IVA initiation (M0) and 24 months after initiation (M24) from all patients **(D)** was performed using the Spearman test and the coefficient of correlation ρ is shown.

During the LUM/IVA treatment, the number of low/non-responders decreased, and the number of patients in both the no decline group and the good responders group increased significantly with time (Figure 6A). After 2 years of treatment, 11/40 (27.5%) were in the no decline group and 22/40 (55.0%) were good responders. Among these 22 patients, 14 (35% of the entire population) had an improvement in ppFEV1 of over 10% (Figure 6A). However, the percentage of good responders was also not significantly different according to the presence or the absence of impaired lung function or BMI *Z*-score < 0 at the LUM/IVA initiation (Figure 6B). Due to the small size of the no decline group and the low/non-responder at M24, we then compared the results of the good responders, defined by their ppFEV1 change >5% at M24, with those of the other patients. In the two groups, there was no change in the BMI *Z*-score in the 2 years prior to the LUM/IVA treatment, but this parameter increased in the 2 years after with a significant improvement at M24 only in the group of good responders (Supplementary Figure 5A). Indeed, the improvement in the BMI *Z*-score was greater in the good responders than in other patients (Supplementary Figure 5B). Surprisingly, there was no significant difference between the two groups of responders in the improvement of sweat chloride (*p* = 0.983), HAV scores (*p* = 0.482), and Bhalla scores (*p* = 0.576). When characteristics assessed at M0 of good responders and the other patients were compared, only the age at the LUM/IVA initiation was significantly different and lower in good responders (Figure 6C and Table 2). Moreover, there was a significant reverse correlation between age at M0 and change in ppFEV1 after 2 years of treatment (rho = −0.33; *p* = 0.04) (Figure 6D), whereas no association was found between the change in ppFEV1 between M0 and M24 and ppFEV1 (rho = −0.09; *p* = 0.57), BMI *Z*-scores (rho = 0.00; *p* = 1.00), and the sweat chloride concentrations (rho = −0.03; *p* = 0.87) at M0, sweat chloride improvement between M0 and M12 (rho = −0.11; *p* = 0.56), or BMI *Z*-score change between M0 and M24 (rho = −0.11; *p* = 0.50).

**Table 2:** Patient characteristics at initiation of lumacaftor/ivacaftor.

	**Other patients**	**Good responders**	***p*1**
*N*	18	22	
Age (years)	14.5 ± 1.67	13.4 ± 1.6	**0.044**
Male sex	10/18 (55.6)	12/22 (54.6)	1.000
Weight *Z*-score	−0.50 ± 1.17	−0.32 ± 0.87	0.514
Height *Z*-score	−0.56 ± 1.10	−0.22 ± 1.03	0.178
BMI *Z*-score	−0.35 ± 1.14	−0.22 ± 0.90	0.888
Past history			
Meconial ileus	1/18 (5.6)	1/22 (4.5)	1.000
DIOS	1/18 (5.6)	0/22 (0.0)	0.450
**At inclusion**			
CFRD	1/18 (5.6)	1/22 (4.5)	1.000
CFLD	4/18 (22.2)	5/22 (22.7)	1.000
*P. Aeruginosa* colonization			
None	9/18 (50.0)	9/22 (40.9)	
Intermittent	2/18 (11.1)	9/22 (40.9)	0.085
Chronic	7/18 (38.9)	4/22 (18.2)	
*S.Aureus* colonization			
None	5/18 (27.8)	3/22 (13.6)	
Intermittent	1/18 (5.6)	2/22 (9.1)	0.587
Chronic	12/18 (66.7)	17/22 (77.3)	
ppFEV1	83.4 ± 13.5	83.2 ± 21.8	0.828
Sweat chloride concentration	103.1 ± 15.4	106.1 ± 18.2	0.592
Ballah score	12.9 ± 3.4	13.7 ± 5.6	0.405
HAV score	3.27 ± 0.46	3.31 ± 0.56	0.965
Treatment			
PERT U/Kg/j			
Proton pump inhibitor	11/18 (61.1)	10/22 (45.5)	0.360
Ursodeoxycolic acid	8/18 (44.4)	8/22 (36.4)	0.748
Laxative treatement	2/18 (11.1)	3/22 (13.6)	1.000
Inhaled antibiotics	10/18 (55.6)	7/22 (31.8)	0.200
**Within the 2 years prior treatment**			
No. of exacerbations	4.0 [2.0; 5.3]	3.0 [1.0; 6.3]	0.913
No. of IV ATB courses	1.0 [0.0; 3.0]	1.0 [0.0; 4.3]	0.865
No. of oral ATB courses	1.5 [0.8; 3.3]	1.5 [0.0; 3.0]	0.677
**First 2 years of LUM/IVA**			
Change in *Z*-score BMI	0.10 ± 0.80	0.44 ± 0.48	0.154
Change in ppFEV1	−4.9 ± 7.9	13.7 ± 7.9	<0.001
No. of exacerbations	4.0 [2.8; 6.3]	3.5 [1.0; 6.0]	0.330
No. of IV ATB courses	2.0 [0.0; 3.3]	0.5 [0.0; 3.5]	0.819
No. of oral ATB courses	2.5 [0.8; 4.0]	1.0 [1.0; 2.3]	0.179

*M0, day of lumacaftor/ivacaftor initiation; ppFEV1, percent predicted forced expiratory volume in 1 s; BMI, body mass index; DIOS, distal intestinal obstruction syndrome; CFRD, cystic fibrosis-related diabetes; CFLD, cystic fibrosis liver disease; P. Aeruginosa, Pseudomonas Aeruginosa; S. Aureus, Staphylococcus Aureus; PERT, pancreatic enzyme replacement therapy*.

*Results are expressed as n/N (%) for categorical variables or as mean ± standard or at median with IQR [median (IQR_25_; IQR_75_) deviation] for quantitative variables. Comparisons between good responders (change in ppFEV1 ≥ 5% after 2 years of treatment by LUM/IVA) and other patients were performed using the Fisher's exact test for categorical variables or the Mann–Whitney test for quantitative variables. A p-value ≤ 0.05 was considered significant*.

### Lumacaftor/Ivacaftor Is Well-Tolerated and Adherence Is Satisfactory

Side effects observed in the 40 patients included were transient chest tightness (*n* = 1), increased anxiety (*n* = 1), transient diarrhea (*n* = 3), and increased aspartate and alanine aminotransferase >5 times the upper limit of normal (*n* = 1). Except for this last patient who temporarily interrupted the treatment, all the others maintained their treatment for at least 2 years independently of the observed response and without any difference between good responders and other patients. No cataract or lens opacities were detected.

Early cessation of treatment (<3 months) occurred in only two patients, due to psychological depression in one case and social difficulties in the other.

## Discussion

This real-life study highlights the long-term effectiveness and safety of LUM/IVA on lung function and nutritional outcomes in CF adolescents in real life. We found a significant improvement in the ppFEV1, from worsening in the 2 years preceding treatment to a significant increase during the 2 years of the LUM/IVA therapy, particularly in patients with ppFEV1 < 80% or BMI *Z*-score < 0 at the LUM/IVA initiation and a concomitant improvement in BMI *Z*-score and sweat chloride concentration after the LUM/IVA treatment.

The impact of LUM/IVA on ppFEV1 appears to be greater in the adolescents in the present study compared with that reported in the pivotal phase III randomized controlled trials, TRANSPORT (+2% to 4% after 24 weeks of LUM/IVA) (17) and PROGRESS (+0.5% at 96 weeks) (8). In the long term, the PROGRESS study demonstrated a slowing down of lung function decline in treated compared with paired non-treated patients from the US registry, but with the persistence of a declining trend in ppFEV1 (8). In comparison, in our study, we showed an increase in ppFEV1 in treated patients. Both the TRANSPORT and PROGRESS studies mainly involved CF adults (8, 17), whereas we focused on CF adolescents only. Our results are consistent with the slowing down of lung function decline after a year of LUM/IVA treatment in adolescents with CF included in the French real-life cohort (18), and with another study performed by Loukou et al. (29). In our study, we confirmed the short term effect at 6 months of LUM/IVA treatment with a proportion of good responders close to a previous report (20). Moreover, we demonstrated both a sustained long-term effect up to 2 years after the LUM/IVA initiation and a significant number of late responders. We also demonstrated that in some patients who were not good responders, ppFEV1 stability was observed, and this represents a satisfactory response since adolescence is the period of greatest decline in lung function. In addition, we demonstrated some benefits in adolescents with impaired lung function or BMI *Z*-score < 0, and at a lesser extent in those with normal ppFEV1 or BMI *Z*-score > 0 at the start of the treatment (i.e., ≈50% of good responders). Patients with impaired lung function or BMI *Z*-score < 0 had also the worst sweat chloride which may be related to a poorer nutritional status or greater CFTR dysfunction. Thus, it was not surprising that restoring CFTR function and/or nutritional status with LUM/IVA has a better effect in these patients. Moreover, we highlighted an inverse correlation between the age at the LUM/IVA initiation and improvement in ppFEV1 after 2 years of treatment. This suggests that early initiation of the treatment at a younger age regardless of pulmonary function or growth parameters may be more beneficial. This was in accordance with the improvement in lung clearance index at 24 weeks of treatment in younger patients (9.1 ± 1.5 years old) with normal lung function as previously described in a 6–11 year phase III study (15).

Interestingly, we observed significant improvement in growth parameters (i.e., weight and BMI *Z*-score), particularly in adolescents with poor BMI *Z*-scores at the start of LUM/IVA as demonstrated in younger patients included in the LUM/IVA phase III (14) or in CF adults (8).

As previously described in an industry-sponsored and larger real-life studies (8, 14, 15, 17–20), we confirmed the improvement induced by LUM/IVA regarding CFTR function, with a significant decrease in sweat chloride concentration, particularly in the most severe patients (low ppFEV1 or BMI). However, we did not find any difference in sweat chloride concentration improvement between responders and non-responders, nor any association between this parameter and ppFEV1 or BMI *Z*-score improvement as previously demonstrated (15, 17, 19, 21).

In contrast, we could not confirm the decrease in pulmonary exacerbation rates and antibiotics consumption in real life in CF adolescents treated with LUM/IVA as found in placebo-controlled and long-term industry sponsored studies (i.e., decrease in exacerbation rates by 30% to 39%) (8, 17) or in a previous real-life study (i.e., decrease in exacerbation rates by 54%) (30). However, in this last study, patients were older (31 ± 11.6 years old) (8, 14, 26) and/or had worse lung function (ppFEV1: 37.4 ± 11.3 %) (30) and/or had a higher rate of exacerbations treated by intravenous antibiotics (3.4 ± 2.4/year) at the time of initiation of LUM/IVA (30) compared with our patients. Moreover, adolescents in our cohort had <2 exacerbations per year, and <1 intravenous antibiotic course per year, thus, the effect of LUM/IVA is more difficult to demonstrate. In agreement with our results, in young adults with F508 homozygous mutations, Tessel et al. found no difference in the annualized rate of exacerbations (31).

We did not find difference in Bhalla (24) scores or HAV (25) scores after a year of treatment as demonstrated in CF adults with advanced disease only (25). However, such results have not been demonstrated in CF children who have a lower level of disease severity. Indeed, CT scans were not included in the industry-sponsored clinical trials of LUM/IVA involving CF children (8, 9, 14–19). In addition, our results are in agreement with the lack of modification in the visual Eichinger score previously demonstrated in CF before and after treatment despite lung clearance index improvement (32). However, a possible lack of sensitivity in the imaging scores may have impacted these results, as the visual scores may not detect a variation of <50% in lung injury due to their scoring systems (32). Nevertheless, it does not reduce the usefulness of treating young patients before irreversible lesions are established. To note, this lack of improvement in imaging scores could also be the consequence of scans performed too early, since the results on ppFEV1 or BMI were only significant after 2 years of treatment in our population.

In our study, the side effects of the treatment were consistent with those observed in the industry-sponsored studies (8, 15, 17) or real-life cohorts (18, 29), and in favor of good tolerability and patient adherence. Interestingly, we observed psychological side effects such as increased anxiety and depression in one patient leading to an early cessation of treatment as previously described in real life (33) but not in the industry-sponsored studies (8, 15, 17).

Our study had several limitations. First, the number of patients included is low compared with other real-life studies and there is no control-group. However, these patients accounted for 84% of our patients eligible for the LUM/IVA treatment in our centers. Second, spirometric tests performed 2 years and 1 year before and after T0 were selected to match the best ppFEV1 obtained at ±1 month from the chosen time point. We use this method to avoid the transient effect of exacerbation on ppFEV1. Third, pre-treatment data were assessed retrospectively. However, such data included standard items, which were uniformly and prospectively recorded during routine follow-up using the same software in the entire South-West network. This data is also channeled to the national registry, decreasing the risk of bias due to missing data. Fourth, only exacerbations requiring antibiotics have been considered, in contrast to less severe pediatric exacerbations due in most instances to viral infections. However, compared with previous clinical trials we assessed exacerbation requiring IV and/or oral antibiotics and not only those requiring IV courses which represent only a subset of antibiotic-treated exacerbations especially in adolescents. Fifth, the adherence to treatment was assessed at each visit but was based on the reports of children or their parents, and this represents an important limit of real-life studies.

## Conclusion

We confirm that in real life, adolescents given long-term LUM/IVA improve their ppFEV1 and BMI *Z*-score, which have been associated with survival in cystic fibrosis. Adolescents with impaired lung function had a greater treatment benefit on lung function, but early introduction appeared to promote better response to the treatment, with good tolerance. In the era of CFTR modulators, real-life studies help the clinicians understand what type of results are really to be expected according to specific age groups.

## Take-Home Message

This real-life study showed short- and long-term benefits of lumacaftor/ivacafor association in adolescents with cystic fibrosis, particularly in the youngest ones, by changing trajectories and improving lung function and nutritional parameters.

## Author's Note

This study focuses on the long-term safety and efficacy of lumacaftor/ivacaftor in children with cystic fibrosis in real-life.

## Data Availability Statement

The raw data supporting the conclusions of this article will be made available by the authors, without undue reservation.

## Ethics Statement

Ethical review and approval was not required for the study on human participants in accordance with the local legislation and institutional requirements. Written informed consent to participate in this study was provided by the participants' legal guardian/next of kin.

## Author Contributions

SB designed and supervised the study, with contributions from AM and MM. SB, AM, LR, FG, CC, RE, JL, MM, GD, MF, and EM included and followed the patients. The statistical analysis was performed by SB, FB, and RE. SB, MM, and AM were major contributors in writing the manuscript and contributed equally to the manuscript. All authors have approved the final manuscript as submitted and agree to be accountable for all aspects of the work.

## Conflict of Interest

SB, FG, CC, RE, FB, and MF conducted clinical trials with Vertex pharmacological agents, on behalf of the European Cystic Fibrosis Society-Clinical Trials Network (ECFS-CTN) and within the scope of ECFS-CTN activities. The remaining authors declare that the research was conducted in the absence of any commercial or financial relationships that could be construed as a potential conflict of interest.

## Publisher's Note

All claims expressed in this article are solely those of the authors and do not necessarily represent those of their affiliated organizations, or those of the publisher, the editors and the reviewers. Any product that may be evaluated in this article, or claim that may be made by its manufacturer, is not guaranteed or endorsed by the publisher.

## Abbreviations

ALT: alanine aminotransferase
AST: aspartate aminotransferase
BMI: body mass index
CFTR: cystic fibrosis transmembrane conductance regulator
HAV: high attenuation volume
ppFEV1: percent predicted forced expiratory volume in 1 s
LUM/IVA: lumacaftor/ivacaftor.
