# Supplementary material for: Long-Term Outcomes in Real Life of Lumacaftor–Ivacaftor Treatment in Adolescents With Cystic Fibrosis
Source: Front Pediatr. 2021 Nov 15;9:744705. doi: 10.3389/fped.2021.744705 (PMC8634876; doi:10.3389/fped.2021.744705)
Supplement: Supplementary file 1 [file Data_Sheet_1.docx]

**ONLINE SUPPLEMENTAL MATERIAL**

**Long-term outcomes of Lumacaftor-Ivacaftor on cystic fibrosis adolescents in real life**

Stéphanie Bui^a*^, MD, Alexandra Masson^b*^, MD, Raphaël Enaud^a,d^ MD-PhD, Léa Roditis^c^, MD, Gaël Dournes^d^, MD, François Galode^a^, MD, Cyrielle Collet^a^, MD, Emmanuel Mas^c^, MD, Jeanne Languepin^b^, MD, Michael Fayon^a,d^, MD-PhD, Fabien Beaufils^a,d,†^, MD, Marie Mittaine^b,†^, MD

* Contributed equally as co-first authors

† Contributed equally as co-last authors

**Affiliations:** ^a^Bordeaux University Hospital, Hôpital Pellegrin-Enfants, paediatric Cystic Fibrosis Reference Center (CRCM), Centre d’Investigation Clinique (CIC 1401), F-33000 Bordeaux, France ; ^b^Limoges University Hospital, paediatric Cystic Fibrosis Reference Center (CRCM), F-87000 Limoges, France ; ^c^Toulouse University Hospital, paediatric Cystic Fibrosis Reference Center (CRCM), Department of Pediatric-pulmonology, F-31059 Toulouse, France; ^d^Bordeaux University, Centre de Recherche Cardio-Thoracique de Bordeaux, U1045, Radiology, F-33000 Bordeaux, France.

**Address correspondence to:** Dr Stéphanie Bui, CRCM pédiatrique, Hôpital Pellegrin-Enfants CHU de Bordeaux, Place Amélie Raba Léon, 33000 Bordeaux, France

*E-mail address*: [stephanie.bui@chu-bordeaux.fr](mailto:stephanie.bui@chu-bordeaux.fr)

**Short title**: Lum-Iva in children, long-term

**Funding Source:** No external funding for this manuscript.

**Financial Disclosure:** The authors have no financial relationships relevant to this article to disclose.

**Conflict of interest:** SB, FG, CC, RE, MF conduct clinical trials with Vertex pharmacological agents, on behalf of the European Cystic Fibrosis Society – Clinical Trials Network (ECFS-CTN) and within the scope of ECFS-CTN activities.

#

**SUPPLEMENTAL FIGURES**

**
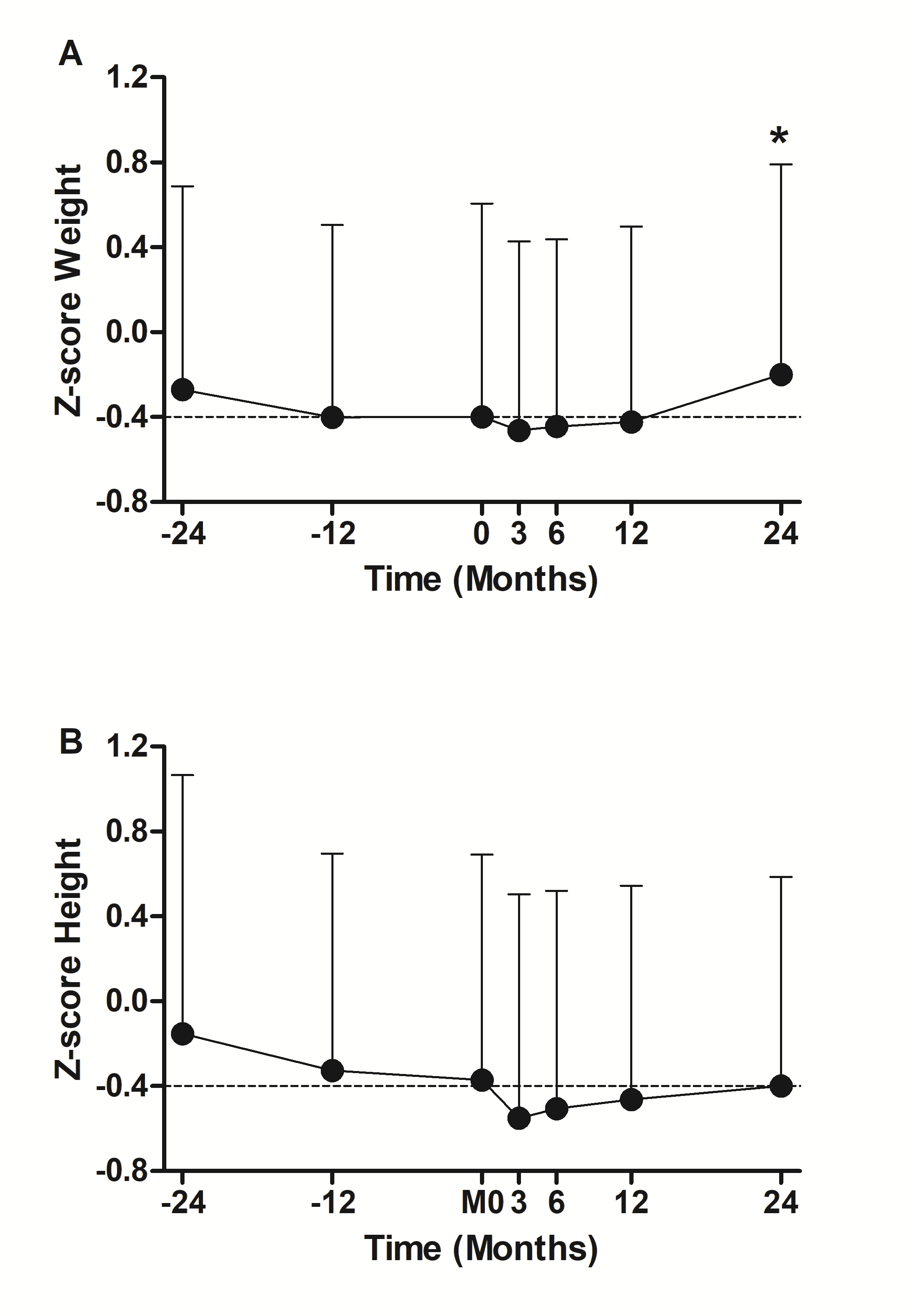
**

**Figure S1. Evolution of Weight and Height Z-scores between two years prior and after LUM/IVA initiation.**

Evolution of Weight (A) and Height (B) Z-scores between two years prior and after LUM/IVA initiation. Data are plotted at each timepoint using all available and were represented at the mean+standard deviation. Comparisons with data obtained at M0 were performed using the Wilcoxon paired test. * p<0.05 compared to M0

**
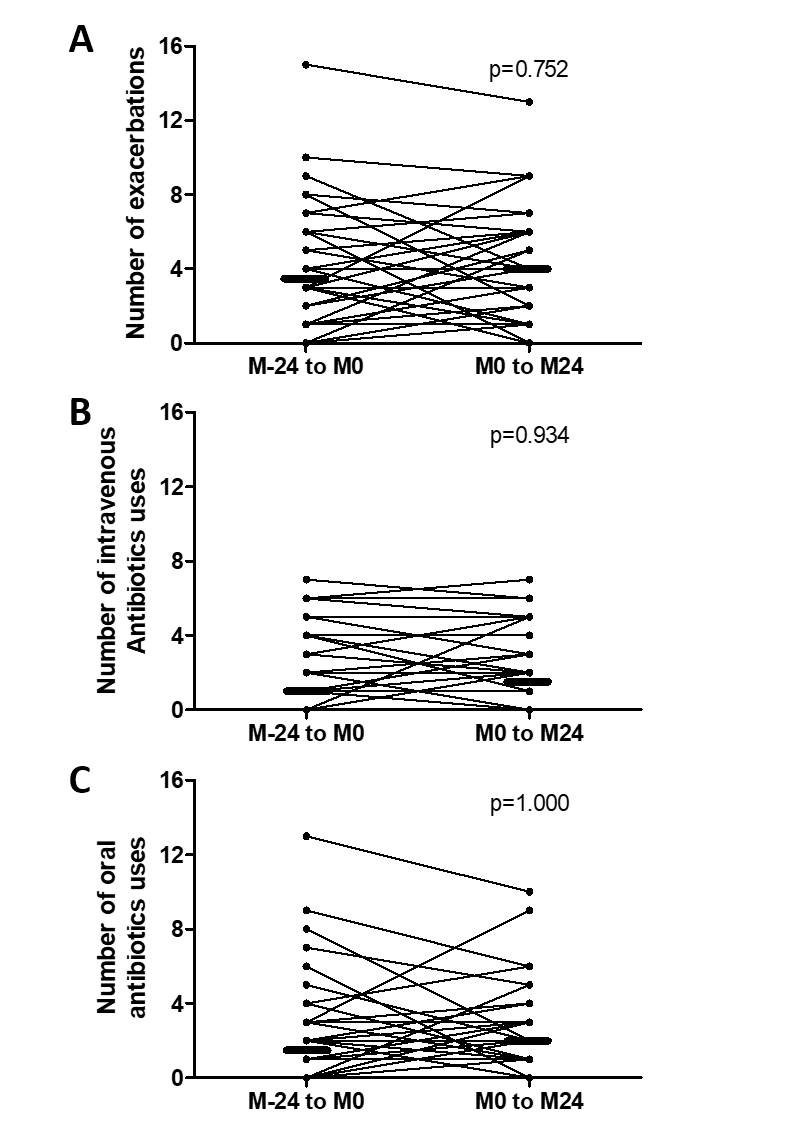
**

**Figure S2. Change in the number of exacerbations or antibiotic use after initiation of Lumacaftor/ivacaftor**

The number of exacerbations (A), intravenous (B) and oral antibiotic courses (C) assessed after two years of treatment by Lumacaftor/ivacaftor (M0 to M24) were compared with those administered in the two years prior to Lumacaftor/Ivacaftor initiation (M-24 to M0). Patient data are presented as individual values (lines) and medians (thick horizontal bars). Comparisons were performed using the Wilcoxon paired test. A *p*-value < 0.05 was considered significant.


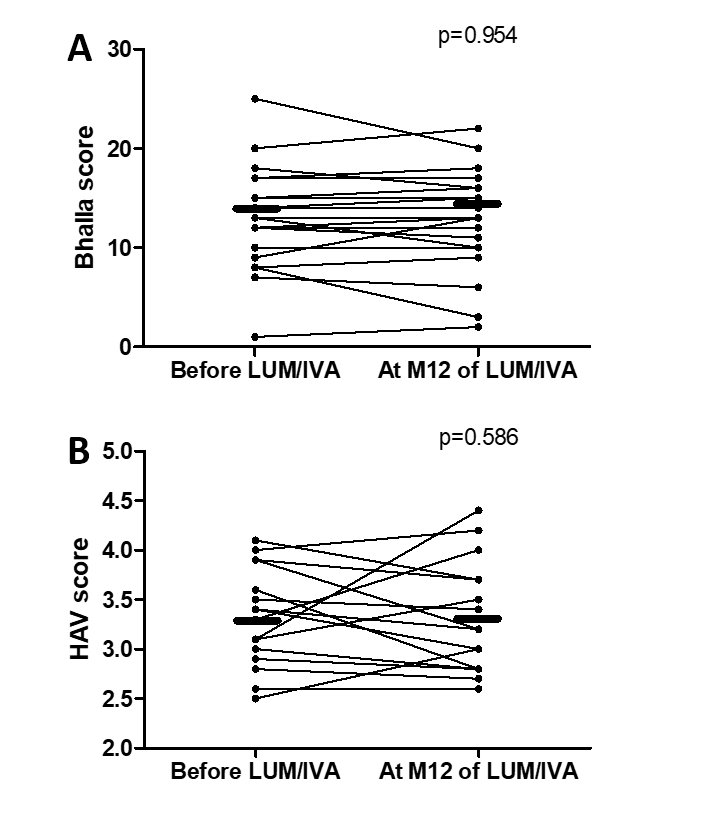


**Figure S3.** **Change in Lung Computed tomography scan scores after initiation of Lumacaftor/ivacaftor**

The Bhalla (A) and the High Attenuation Volume (HAV)scores (B) assessed after 12 months of Lumacaftor/ivacaftor treatment (M12) were compared to scores performed prior to Lumacaftor/ivacaftor treatment. Patient data are presented as individual values (lines) and medians (thick horizontal bars). Comparisons were performed using the Wilcoxon paired test. A *p*-value < 0.05 was considered significant.


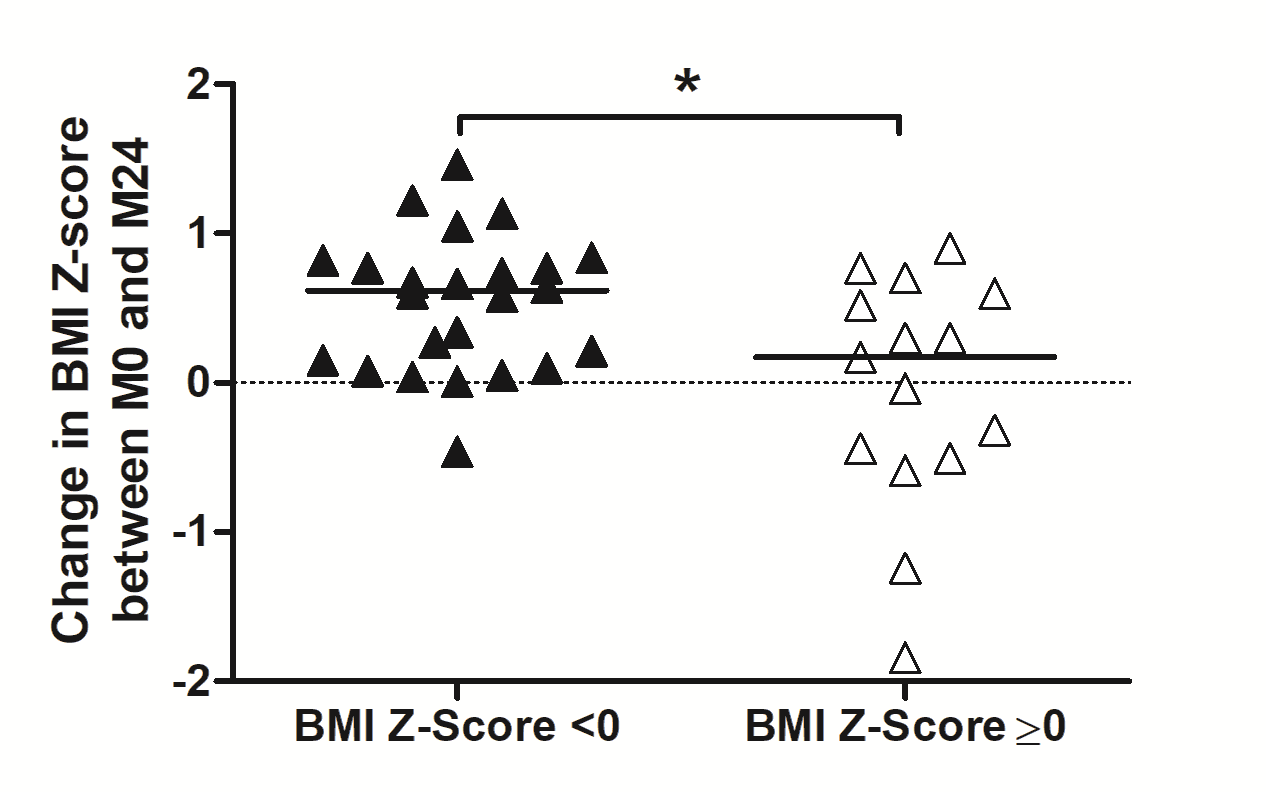


**Figure S4. Change in BMI Z-score between LUM/IVA initiation (M0) and 2 years after (M24) according to BMI Z-score at M0**

Change in BMI Z-score between M0 and M24 in patients with (black triangle) or without (empty triangle) BMI Z-score < 0 at M0. Comparison was performed using Mann-whytney test (*p <0.05)


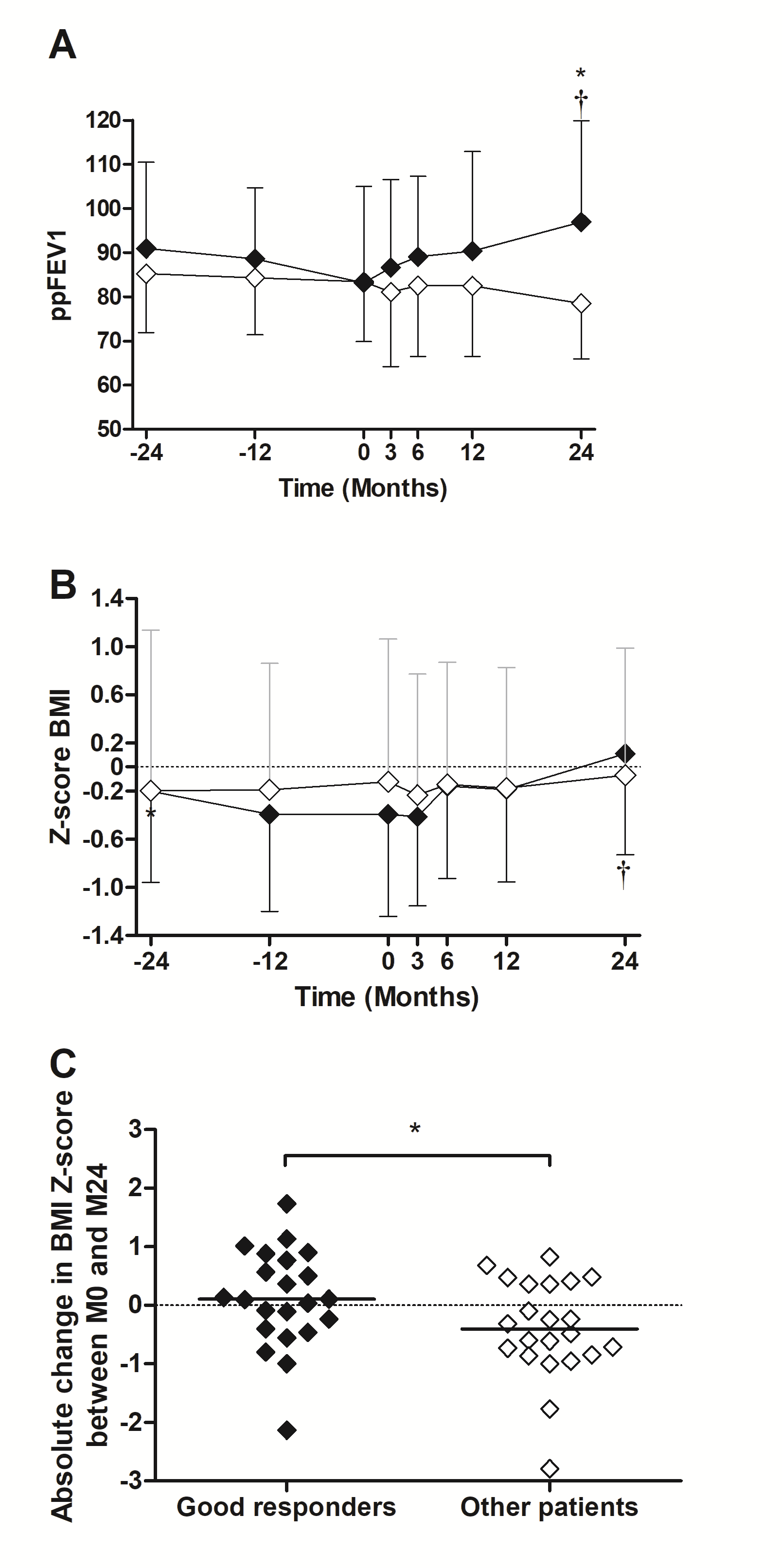


**Figure S5. Change in ppFEV1 and BMI Z-score in good responders and other patients**

Evolution of ppFEB1 (A) and BMI Z-score (B) between two years before (M-24) and two years after (M24) LUM/IVA initiation (M0) in good responders (ppFEV1 increase > 5% between M0 and M24, black diamond) and other patients (non-decline group (ppFEV1 increase between 0 and 5% between M0 and M24) and low / non responders ((ppFEV1 decrease between M0 and M24), empty diamond). Change in BMI Z-score between M0 and M24 in good responders and other patients (C). Comparisons with M0 were performed using Wilcoxon paired test and ^†^ indicates significant difference (p<0.05) compared to M0 in good responders. Comparisons between groups were performed using Mann-Whitney test (*p <0.05).
